# Fusion Oncogenes Are Associated With Increased Metastatic Capacity and Persistent Disease in Pediatric Thyroid Cancers

**DOI:** 10.1200/JCO.21.01861

**Published:** 2022-01-11

**Authors:** Aime T. Franco, Julio C. Ricarte-Filho, Amber Isaza, Zachary Jones, Neil Jain, Sogol Mostoufi-Moab, Lea Surrey, Theodore W. Laetsch, Marilyn M. Li, Jessica Clague DeHart, Erin Reichenberger, Deanne Taylor, Ken Kazahaya, N. Scott Adzick, Andrew J. Bauer

**Affiliations:** ^1^ Division of Endocrinology and Diabetes, Children's Hospital of Philadelphia, Philadelphia, PA; ^2^ Division of Oncology, Children's Hospital of Philadelphia, Philadelphia, PA; ^3^ Department of Pathology and Laboratory Medicine, Children's Hospital of Philadelphia, Philadelphia, PA; ^4^ School of Community and Global Health, Claremont Graduate University, Claremont, CA; ^5^ Department of Biomedical and Health Informatics, Children's Hospital of Philadelphia, Philadelphia, PA; ^6^ Division of Pediatric Otolaryngology, Children's Hospital of Philadelphia, Philadelphia, PA; ^7^ Department of Surgery, Children's Hospital of Philadelphia, Philadelphia, PA; 8 Deceased.

## Abstract

In 2014, data from a comprehensive multiplatform analysis of 496 adult papillary thyroid cancer samples reported by The Cancer Genome Atlas project suggested that reclassification of thyroid cancer into molecular subtypes, *RAS*-like and *BRAF*-like, better reflects clinical behavior than sole reliance on pathologic classification. The aim of this study was to categorize the common oncogenic variants in pediatric differentiated thyroid cancer (DTC) and investigate whether mutation subtype classification correlated with the risk of metastasis and response to initial therapy in pediatric DTC.

**METHODS:**

Somatic cancer gene panel analysis was completed on DTC from 131 pediatric patients. DTC were categorized into *RAS*-mutant (*H-K-NRAS*), *BRAF*-mutant (*BRAF* p.V600E), and *RET*/*NTRK* fusion (*RET*, *NTRK1*, and *NTRK3* fusions) to determine differences between subtype classification in regard to pathologic data (American Joint Committee on Cancer TNM) as well as response to therapy 1 year after initial treatment had been completed.

**RESULTS:**

Mutation-based subtype categories were significant in most variables, including age at diagnosis, metastatic behavior, and the likelihood of remission at 1 year. Patients with *RET*/*NTRK* fusions were significantly more likely to have advanced lymph node and distant metastasis and less likely to achieve remission at 1 year than patients within *RAS-* or *BRAF*-mut subgroups.

**CONCLUSION:**

Our data support that genetic subtyping of pediatric DTC more accurately reflects clinical behavior than sole reliance on pathologic classification with patients with *RET*/*NTRK* fusions having worse outcomes than those with *BRAF*-mutant disease. Future trials should consider inclusion of molecular subtype into risk stratification.

## BACKGROUND

In 2014, The Cancer Genome Atlas (TCGA) reported that classification of adult papillary thyroid cancer (PTC) into molecular subtypes on the basis of an messenger RNA expression signature, *RAS*-like and *BRAF*-like, more accurately reflected cellular signaling, cellular differentiation, and clinical behavior when compared with histology alone.^1^ This observation has led to discussions as to whether identification of oncogenic alterations could be used to stratify therapy, including the extent of surgery, lobectomy versus total thyroidectomy, as well as central compartment lymph node dissection.^2,3^

**CONTEXT**

**Key Objective**
The incidence of regional and distant metastasis is higher in pediatric patients compared with adults with differentiated thyroid cancer (DTC). Does the adult two-tiered oncogene classification paradigm into *RAS*-like versus *BRAF*-like tumors similarly predict phenotypic behavior and outcomes in pediatric DTC (PedDTC)?
**Knowledge Generated**
This study showed that *RET* and *NTRK* fusions are the most prevalent genetic alterations in PedDTC. Patients with *RET/NTRK* fusion had the highest risk of metastases and were less likely to achieve remission at 1 year postsurgery. A 3-tiered classification of *RAS*-mutant versus *BRAF*-mutant versus *RET/NTRK* fusions more accurately correlates with phenotypic behavior and outcomes in PedDTC.
**Relevance**
Our results provide greater clarity into the oncogenic alterations in PedDTC that confer the greatest risk for metastasis and persistent disease. These findings highlight the need to further define differences in the molecular landscape between pediatric and adult DTC to optimize clinical care and use of molecularly targeted therapies.

With the reduced costs of next-generation sequencing (NGS), the use of somatic cancer gene panel analysis in pediatric patients with differentiated thyroid cancer (DTC) has expanded with current data showing a shifted distribution of driver alterations with a higher incidence of oncogenic fusions rather than point mutations in children and adolescents compared with adults.^4,5^ Similar to the TCGA-based molecular subtype classification system on the basis of messenger RNA expression signatures, we sought to determine whether oncogenic subtyping on the basis of identified mutations or fusions predicts phenotypic behavior and outcomes in pediatric patients with DTC.

## METHODS

### Patient Population

Our series comprised 131 pediatric thyroid tumors (122 PTCs and nine follicular thyroid carcinoma [FTCs]) from surgical specimens. This included 66 surgical specimens sequentially collected from 2016 to 2019 and analyzed in the Department of Pathology at Children's Hospital of Philadelphia (CHOP) as well as 65 archived surgical specimens collected from 1989 to 2012 that were previously genotyped on a commercial platform.^6^ All of the tumors were sequentially collected and analyzed to limit selection bias because of sample adequacy. The research protocol was approved by CHOP's Institutional Review Board (IRB). All samples used for data analysis were approved by our institute IRB (local human investigations committee). A waiver of consent was granted by the CHOP IRB for all surgical specimens included in the study. We were not required to file an assurance with the Department of Health and Human Services.

DTC classification was based on standard histopathologic criteria defined by the WHO.^7^ Tumors were staged according to the 7th edition of the American Joint Committee on Cancer (AJCC) staging manual.^8^ Historic samples were reassessed to these same criteria when possible, on the basis of data availability. Invasion was defined as spread to regional lymph nodes and/or distant metastasis. Remission was assessed at 1 year ± 3 months after surgery. Remission was defined as a basal thyroglobulin (Tg) and antithyroglobulin below the lower limit of detection and no evidence of persistent thyroid cancer on radiologic imaging and/or radioiodine whole-body scan (RAI-WBS). Neck ultrasound was used for patients with disease limited to the neck and undetectable Tg. Chest computed tomography was added for patients with a history of pulmonary metastasis on initial imaging. RAI-WBS was used to assess for persistent disease in patients with detectable Tg (> 10 ng/mL and/or increasing trend) and no evidence of persistent thyroid cancer on the basis of neck ultrasound and chest computed tomography. All patients undergoing thyroidectomy with confirmed malignancy were placed on levothyroxine suppressive therapy targeted to achieve a thyroid stimulating hormone below the lower end of the normal range, < 0.5 mIU/L.

### Sequencing Platform and Variant Calling

The 66 tumors collected between 2016 and 2019 were sequenced as part of routine clinical care using both the CHOP Solid Tumor Panel (CSTP) and CHOP Cancer Fusion Panel (CCFP). CSTP is a targeted NGS assay encompassing 238 cancer genes. The assay is designed to detect single-nucleotide variants (SNVs), indels, and copy-number alterations (CNAs) as described previously.^9^ Briefly, genomic DNA was extracted from the tumor samples, and libraries were prepared using probes targeting 238 genes and sequenced on HiSeq platform using 150 bp paired-end sequencing. Sequence data were analyzed using the institutional software ConcordS v2 (for SNVs and indels)^9^ and NextGENe v2 NGS Analysis Software (for CNAs; SoftGenetics, LLC, State College, PA). Fusion gene detection was performed using the CHOP Cancer Fusion Panel as previously described.^10^ Briefly, target-specific primers covering 673 exons were custom-designed to identify known fusion genes and potential novel fusion genes associated with 110 cancer genes using Anchored Multiplex PCR (AMP) technology (ArcherDX, Inc Boulder, CO). The 65 cases collected between 1989 and 2012 had been previously genotyped using Asuragen's first-generation thyroid test, miRInform Thyroid Test, as described.^6^ This panel interrogates the presence of the most common mutations in *BRAF*, *HRAS*, *KRAS,* and *NRAS,* and three fusion transcripts (*RET*/*PTC1*, *RET*/*PTC3*, and *PAX8*/*PPARG*). The miRInform panel did not include analysis for *DICER1* mutations, *NTRK* fusions, or any novel *RET* partners. Unfortunately, tissue from the miRInform cohort was not available for repeat analysis using the more comprehensive CHOP panels. All cancer genes included in the CSTP and CCFP panels and the miRInform Thyroid Test are listed in the Data Supplement (online only).

Mutations were subcategorized into three groups, *RAS*-mutant (*H*/*K*/*NRAS* mutations and *PAX8*/*PPARG* fusions), *BRAF*-mutant (*BRAF* p.V600E mutations), or *RET*/*NTRK* fusions (*RET*, *NTRK1*, and *NTRK3* fusions) on the basis of previous published reports in adults^1,2,11^ as well as pediatric data showing genotype-associated differences in invasive behavior.^12-14^ Tumors with no identified genetic alteration in the miRInform Thyroid Test or CSTP panel, or a genetic alteration identified by the CSTP panel that has not been previously identified as a driver mutation in pediatric DTC (PedDTC), were characterized as indeterminate.^9^

### Data Analyses

Data analyses were performed using R 4.0.5 and R Studio 1.4.1106 (RStudio, PBC). Frequencies and proportions were used as descriptive statistics for categorical variables. Mutation status was the primary variable of interest and so, this was explored over a number of different dimensions of the data. Associations between covariates and mutation status were tested using Fisher's exact tests, to account for small cell sizes. A two-sided *P* value of < .05 was considered statistically significant. Mutation data and clinicopathologic characteristics from adult PTC were collected from the TCGA Data Portal.^15^ Comparisons between adult and pediatric variables were tested using Fisher's exact tests.

## RESULTS

### Patient Demographics and Thyroid Pathology

Clinicopathologic characteristics of the study population are summarized in Table 1 and the Data Supplement. The study cohort included 131 samples from 97 (74%) female patients and 34 (26%) male patients with a mean age of 14.53 ± 2.99 years. Tumors were divided on the basis of their dominant histopathologic features: 72 (55%) classic variant (cPTC), 31 (23.7%) follicular variant (fvPTC), 14 (10.7%) diffuse sclerosing variant (dsvPTC), 5 (3.7%) were other forms of PTC, including cribriform-morular variant (cmvPTC), solid variant (svPTC), and Warthin-like (WLPTC), and 9 (6.9%) FTC.

**TABLE 1.**
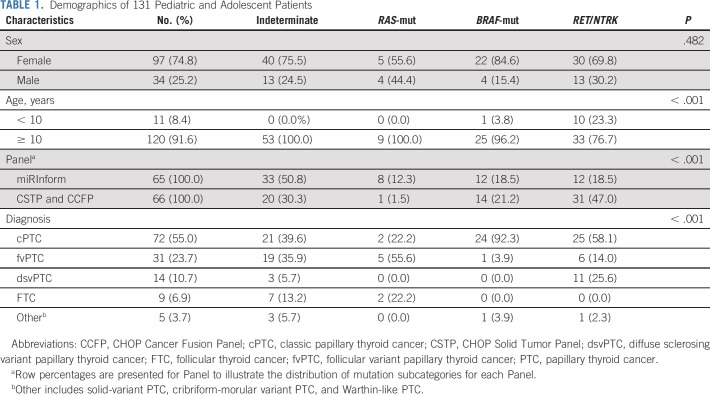
Demographics of 131 Pediatric and Adolescent Patients

### Identification and Grouping of Genetic Alterations.

Of the 131 patient samples, genetic alterations were identified in 78 of the 131 tumors; nine with an *RAS*-mutant (6.9%, 9 of 131), 26 with *BRAF* (19.8%, 26 of 131), and 43 with an *RET* or *NTRK* fusion (32.8%, 43 of 131; Table 1, Data Supplement). *RAS*-mutant, *BRAF*, and *RET*/*NTRK* fusions were mutually exclusive events in every tumor analyzed. On the basis of previous reports, *RET* fusions and *NTRK* fusions were hypothesized to confer a similar risk for malignancy, and correlate with similar risk of metastatic behavior, and were therefore grouped together in the analysis.^12-14^ There were 53 tumors classified as indeterminate. Secondary to more limited coverage of oncogenic driver alterations, there were more tumors classified as indeterminate in the miRInform group compared with the CSTP and CCFP group (50.8% *v* 30.3%); most notably, fewer *RET*/*NTRK* fusions were identified in the miRInform group compared with the CSTP and CCFP group (18.5% *v* 47.0%).

**FIG 1.**
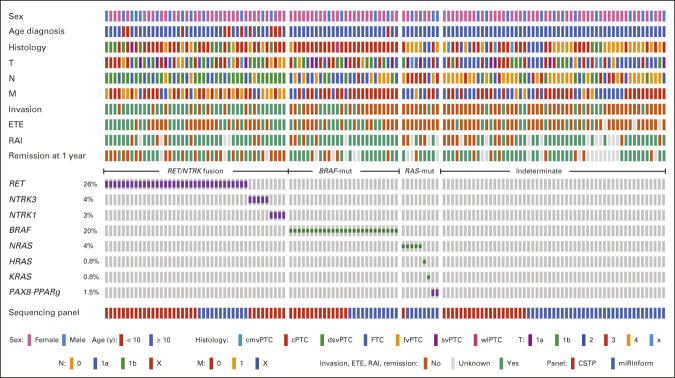
Genetic landscape and clinicopathologic characteristics of 131 pediatric thyroid cancers. Clinicopathologic characteristics include sex, age, histology, T status, N metastasis status, M, invasion, ETE, RAI therapy, and remission. The most frequent genetic alterations (fusion oncogenes and mutations) and their prevalence are shown. Genetic alterations were categorized into *RET*/*NTRK* fusions (*RET* and *NTRK1*/*3* fusions), *BRAF*-mut (*BRAF* p.V600E), and *RAS*-mut (*H-K-NRAS* and *PAX8*/*PPARG*). cmvPTC, cribriform-morular variant papillary thyroid cancer; cPTC, classic papillary thyroid cancer; CSTP, CHOP Solid Tumor Panel; dsvPTC, diffuse sclerosing variant papillary thyroid cancer; ETE, extrathyroidal extension; FTC, follicular thyroid cancer; fvPTC, follicular variant papillary thyroid cancer; M, distant metastasis status; N, lymph node; RAI, radioactive iodine; svPTC, solid variant papillary thyroid cancer; T, tumor status; WLPTC, Warthin-like papillary thyroid cancer.

Figure 1 summarizes the mutational landscape of PedDTC and clinicopathologic features. As previously reported, we found a correlation between genotype and pathologic phenotype.^12-14^ *RAS* mutations and *PAX8-PPARG* fusions were more commonly associated with fvPTC and FTCs than the other genotypes. There were four *NRAS* p.Q61R and one *PAX8-PPARG* fusion in five encapsulated fvPTC, one *NRAS* p.Q61R in a cPTC, one *PAX8*/*PPARG* in a cPTC/fvPTC/svPTC mixed histology, and a single *HRAS* p.Q61R and single *KRAS* p.G12V in two FTC samples. The *BRAF* p.V600E mutation was most commonly associated with cPTC, observed in 26 (19.8%) samples. *RET*/*NTRK* fusions were found in 43 (32.8%) tumor samples spread across various subtypes of PTC, including 22 cPTC samples, 11 dsvPTC, five fvPTC, one svPTC, one fvPTC/svPTC, one cPTC/fvPTC, and two cPTC/fvPTC/svPTC. The fusions included 20 *RET*/*PTC1* (*CCDC6-RET*), one *RET*/*PTC2* (*PRKAR1A-RET*), eight *RET*/*PTC3* (*NCOA4-RET*), two *SPECC1L-RET*, one *EML4-RET*, one *TRIM24-RET*, one *CCDC186-RET*, two *TPR-NTRK1*, one *IRF2BP2-NTRK1*, one *SQSTM1-NTRK1*, and five *ETV6-NTRK3*.

Of note, four additional potential kinase-activating in-frame fusions (two *TFG-MET*, one *TG-FGFR1*, and one *PRKD2-BRAF*) and three mutations (one *BRAF* p.T599del and two *TSHR* p.M453T and p.D633Y) were identified by the more comprehensive CSTP and CCFP panel. In addition, we found mutations associated with increased risk of thyroid cancer: three cases harbored alterations of *APC* (three cmvPTC) and two cases harbored biallelic mutations of *DICER1* (one fvPTC and one FTC). The 12 cases above were classified as indeterminate because of their low prevalence and uncertain molecular category. All genetic drivers found in this study are listed in the Data Supplement.

### Relationship Between Genetic Alterations and Clinical Characteristics

**FIG 2.**
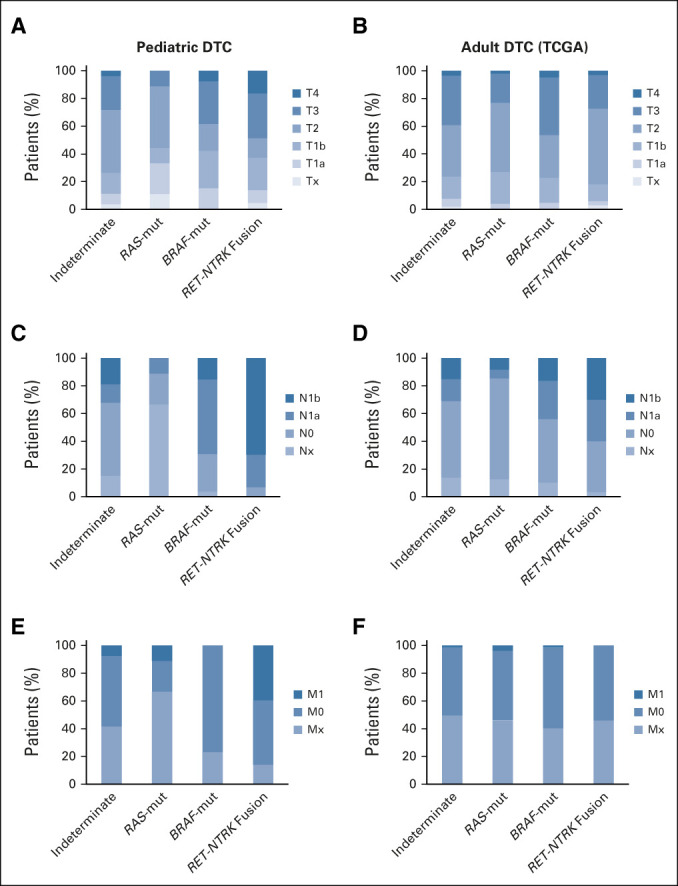
TNM staging of pediatric and adult thyroid cancers according to mutational status. (A and B) Tumor size, (C and D) lymph node metastasis, and (E and F) distant metastasis classification of pediatric DTC (N = 131; combined CSTP and miRInform samples) and adult DTCs (N = 496; TCGA) categorized by indeterminate, *RAS*-mut, *BRAF*-mut, and *RET*/*NTRK* fusion variants. CSTP, CHOP Solid Tumor Panel; DTC, differentiated thyroid cancer; M, distant metastasis status; N, lymph node; T, tumor status; TCGA, The Cancer Genome Atlas.

The genetic alterations and correlation with clinicopathologic characteristics and outcomes are summarized in Table 2. Several strong associations between the covariates and mutation categories were observed. When comparing the four categories of mutation status: indeterminate, *RAS*-mutant, *BRAF*-mutant, and *RET*/*NTRK* fusion, all variables were found to be statistically significant (*P* value < .05), except for sex and AJCC T (tumor size) category. There were very few *RAS*-mutant samples; therefore, we restricted analysis and comparison to *BRAF*-mutant and *RET*/*NTRK* fusions. After restricting the sample to those with *BRAF*-mutant versus *RET*/*NTRK* fusion status, statistically significant associations were still found among age, AJCC N (lymph node metastasis) and M (distant metastasis, all pulmonary) categories as well as remission at 1 year (*P* value < .05). Significantly, no distant metastasis was detected in any patients with *BRAF*-mutant thyroid tumors. Forty-two percent of patients with *RET*/*NTRK* fusion achieved remission at 1 year compared with 61.5% of patients with tumors harboring a *BRAF* mutation (Fig 2). Three patients with no remission at 1 year, subsequently achieved remission at a later date (one *RET/NTRK* fusion and two indeterminate). No mortality was observed in any patients.

**TABLE 2.**
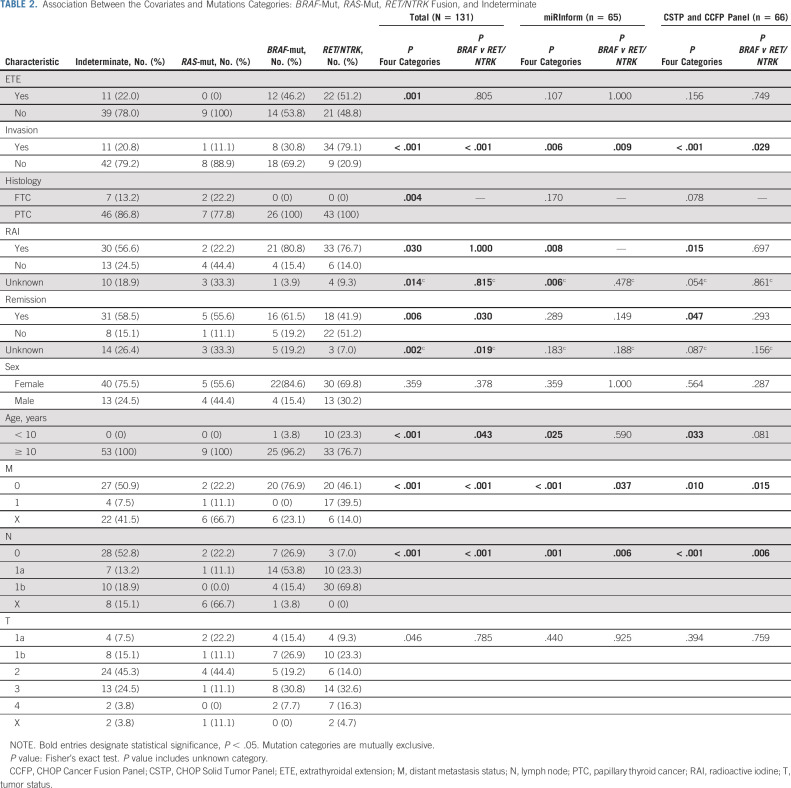
Association Between the Covariates and Mutations Categories: *BRAF*-Mut, *RAS*-Mut, *RET/NTRK* Fusion, and Indeterminate

cPTC variant was the most common histology for both *BRAF*-mut (n = 24) and *RET*/*NTRK* fusion subgroups (n = 25; 16 *RET*, three *NTRK1*, and three *NTRK3* fusions). Even within this histologic variant, we observed significant differences in metastatic behavior and remission status between these molecular subgroups. Metastases to lateral neck lymph nodes were found in 4 of 24 (17%) of patients with a *BRAF* mutation and 15 of 25 (60%) of patients with *RET*/*NTRK* fusions. Distant metastasis was present in 9 of 25 (36%) of the *RET*/*NTRK* fusion subgroup and none of the patients within the *BRAF*-mut subgroup. Moreover, persistent disease at 1 year was more frequent in the subgroup harboring *RET*/*NTRK* fusions (9 of 36; 36%) than those with mutations in *BRAF* (4 of 24; 17%; Data Supplement).

Consistent with previously published results, a greater percentage of pediatric patients presented with nodal and distant metastasis compared with adult tumors reported in the TCGA database (Fig 2).^16^ In adult DTC samples, lateral lymph node metastasis was slightly increased in patients with *RET*/*NTRK* fusions versus *BRAF*-mut, but the difference is less pronounced than in our pediatric population (Fig 2). By contrast, adults harboring *RAS*-mutant PTC rarely present with lymph node or distant metastasis.^17^ This also seems to be the case for pediatric patients with *RAS*-mutant DTC, although the significance of this is unknown because of the limited number of *RAS*-mutant DTCs in our cohort.

*RET*/*NTRK* fusions were more common in our cohort of patients age < 10 years compared with patients age > 10 years, as previously reported.^13^ Ten out of 11 (91%) patients age < 10 years harbored fusion events. The prevalence gradually decreased in pediatric patients age older than 10 years (27%) and into adulthood (9%). By contrast, only one *BRAF* p.V600E mutation (9%) was found among patients age < 10 years compared with 25 pediatric patients age ≥ 10 years (20%) with further increased prevalence in adult patients with *BRAF* mutations, where 58% of PTC in adults harbor a *BRAF* mutation (Table 1 and the Data Supplement).

## DISCUSSION

The thyroid Cancer Genome Atlas reported that classification of adult PTC into molecularly defined groups, *RAS*-like and *BRAF*-like, more accurately reflects cellular signaling, cellular differentiation, and clinical behavior when compared with histology alone.^1^ Our data suggest that separating pediatric PTC with *BRAF* p.V600E mutations from PTC with *RET*/*NTRK* fusions more closely aligns with clinicopathologic features with *BRAF*-positive PTC less common with decreased age and *RET*/*NTRK* fusion–positive PTC more commonly associated with lateral neck (N1b) and distant (M1, pulmonary) metastasis. Both *BRAF* and *RET*/*NTRK* fusions were identified in cPTC; however, even within the same pathologic variant, the molecular driver more accurately predicted metastatic behavior (Data Supplement). This observation is in keeping with previous reports in adults^1,2,11^ and pediatrics^12,13^ that reclassification of thyroid cancers into genetic and molecular subtypes provides an opportunity for better informed clinical management compared with pathologic classification alone. *RAS*-mutants showed similar predictability, associated with reduced metastatic behavior in our pediatric cohort compared with published data in adults; however, the low incidence of *RAS*-mutants in our cohort prevented clinically relevant statistical analysis.

There are differences in the composition of genetic variants in the pediatric population when compared with the adult. The incidence of a *BRAF* p.V600E mutation in pediatric patients with PTC is lower and there is a lower risk for *BRAF*-associated widely invasive disease with decreased radioactive iodine avidity.^5,12,13^ In addition, coexisting mutations in the *TERT* promoter, *TP53*, and genes encoding effectors of the PI3K pathway (*PIK3CA*) are frequent in *BRAF*-mut (approximately 10%) and *RET/NTRK* (approximately 2.5%) advanced adult thyroid tumors.^1^ In our cohort of pediatric tumors screened by the CSTP panel (n = 66), we did not find any coexisting mutations in *BRAF*-mut or *RET*/*NTRK* tumors. This suggests that with increasing age, other age-related host factors may result in the accumulation of additional genetic alterations that negatively influence differentiation, response to therapy, and, subsequently, disease-specific morbidity and mortality supporting separation of how oncogenic landscape data are interpreted and incorporated into clinical practice for pediatric versus adult patients with DTC.

The availability of the CSTP has provided us with a wider lens in which to view pediatric thyroid carcinoma. The incorporation of a comprehensive cancer gene panel lowered the number of samples without an identifiable alteration and led to discovery of several findings that warrant further investigation. We demonstrate that 50% of tumors with distal metastasis had indeterminate drivers using the miRInform panel. By contrast, only 30% of tumors characterized with the more comprehensive CSTP panel had indeterminate drivers and within these 20 samples, 12 harbored mutations that likely have an important role in thyroid tumorigenesis. These 12 cases were included in the indeterminate subgroup because of their relatively low prevalence and uncertain molecular category. These included fusions *PRKD2-BRAF*, *TFG-MET* (n = 2), and *TG-FGFR1*, and mutations *TSHR* p.M453T and p.D633Y and *BRAF* p.T599del, all previously reported in thyroid tumors with the exception of the novel *PRKD2-BRAF* fusion (Data Supplement).^1,18-20^ Future studies are underway to more clearly define the influence of these genetic alterations on altered signaling pathways and thyroid cell differentiation.

The observation of a higher incidence of *RET*/*NTRK* fusions as well as their association with more metastatic behavior compared with *BRAF* in our pediatric cohort emphasizes the importance of expanding our knowledge of the PedDTC molecular landscape. In the adult-based TCGA, analysis revealed a fairly quiet adult PTC genome allowing for a more precise evaluation of the effects of the genetic drivers on signaling pathway activation and differentiation. In the TCGA, a 71-gene signature generated by comparison of *BRAF*-mut and *RAS*-mut tumors was used to construct a *BRAF*^V600E^*-RAS* score (BRS) that separated tumors on the basis of MAPK pathway output and clinical behavior. In the TCGA analysis, *NTRK1*/*3* fusions were largely neutral, and virtually all *RET* fusions were only weakly *BRAF*-like.^1^ The *RET-*fusion subgroup in these adult tumors also exhibited an intermediate Thyroid Differentiation Score (TDS; 16-gene signature including thyroid specific genes such as *SLC5A5*, *TG*, *TPO*, *PAX8*, *TSHR,* and others), lower than the well-differentiated *RAS*-mut (*H*/*K*/*NRAS* and *PAX8*/*PPARG*) tumors and higher than the *BRAF*-mut (*BRAF* p.V600E; Data Supplement). Considering the high prevalence of *RET*/*NTRK* fusions in PedDTC, and their association with more metastatic behavior, it will be crucial to generate the transcriptional signatures of *RET*/*NTRK* and *BRAF-*mutant subgroups in the pediatric population to understand the differential impact of these alterations on signaling pathways, differentiation, and clinical outcomes.

There are several limitations to this study, supporting the need for multicenter, prospective studies to expand our knowledge on the potential use of genetic and molecular analysis for stratification of therapy. The sample size is relatively small at 131 samples, especially as the analysis was divided between the miRInform and CSTP samples at 65 and 66 samples, respectively. It is also worth repeating that *RET*/*NTRK* prevalence in this study may be underestimated as the miRInform panel did not include analysis for *NTRK1*, *NTRK3*, *BRAF*, and *ALK* fusions as well as expanded *RET* fusion isoforms. Unfortunately, as previously stated, the samples initially analyzed by the miRInform panel were not available for reanalysis using the CSTP. Our conclusions are only on the basis of oncogenic driver alterations. Additional studies are ongoing to define the differentiation score on the basis of multiplatform analysis including RNA and microRNA expression. Finally, we demonstrated a significant association between oncogenic driver and remission at 1 year postsurgery. Although disease progression was rare in this cohort of patients, given the indolent nature of thyroid cancer in children, additional longitudinal data are needed to determine whether oncogenic driver will also be associated with long-term disease-free survival and/or risk of disease progression.

An important strength of our study is that many of these oncogenic events that are more common in pediatrics compared with adults with DTC can now be targeted with FDA-approved agents. Of particular note is the identification of *RET* and *NTRK* fusions and positive association of these events with persistent disease and metastatic spread. Larotrectinib (NTRK inhibitor) and selpercatinib (RET inhibitor) have shown dramatic efficacy in clinical trials in both solid tumors and hematologic malignancies, including a limited number of thyroid tumors harboring *NTRK* and *RET* fusions included in these studies. Significantly, these studies have shown durable responses in a multitude of patients with limited adverse events.^21,22^ Interestingly, larotrectinib was shown to induce partial response and restore iodine uptake in one case of RAI refractory adult thyroid cancer harboring the *EML4-NTRK3* fusion,^23^ opening the way for similar therapies in pediatric tumors where these alterations are frequent and often associated with poor prognosis. This provides an opportunity for collaboration between oncologists and endocrinologists to better define the etiology of thyroid tumor progression, and shift the treatment paradigm to molecularly targeted therapies for patients with greatest risk of persistent, metastatic disease.

In conclusion, the combined data set used in this study represents an evolutionary change in the information gained from genetic and molecular analysis over the span of a few short years. On the basis of our data, categorizing PedDTC into *RAS*-mutant, *BRAF*-mutant, and *RET*/*NTRK* fusion variants more accurately separates the higher risk of invasive behavior for *RET*/*NTRK* fusion–driven PTC compared with PTC harboring *BRAF* p.V600E mutations. *RET*/*NTRK* fusion tumors metastasize to lateral neck lymph nodes at a significantly higher frequency than *BRAF* p.V600E PTC. Furthermore, we did not observe distant metastasis in any patients with *BRAF* p.V600E mutations. These findings support the incorporation of somatic cancer gene analysis to improve the diagnostic accuracy for fine needle aspiration as well as the potential utility to incorporate oncogenic data to stratify the surgical approach and to identify tumors that may benefit from oncogene-specific systemic therapies. Additional studies are underway to define the differences in differentiation score between *BRAF* p.V600E and *RET/NTRK* fusion PTC, differences between pediatric and adults PTC with the same oncogenic alterations, as well as to confirm the tumorigenic potential of the novel alterations identified using the comprehensive CSTP panel.

## PREPRINT VERSION

Preprint version available on bioRxiv (https://www.biorxiv.org/content/10.1101/2021.07.23.453235v1).

## References

[1] Cancer Genome Atlas Research Network: Integrated genomic characterization of papillary thyroid carcinoma. Cell 159:676-690, 2014 25417114 10.1016/j.cell.2014.09.050 PMC4243044

[2] Krasner JR Alyouha N Pusztaszeri M : Molecular mutations as a possible factor for determining extent of thyroid surgery. J Otolaryngol Head Neck Surg 48:51, 2019 31623671 10.1186/s40463-019-0372-5 PMC6796357

[3] Labourier E Fahey TJ III: Preoperative molecular testing in thyroid nodules with Bethesda VI cytology: Clinical experience and review of the literature. Diagn Cytopathol 49:E175-E180, 2021 33052631 10.1002/dc.24637 PMC7983887

[4] Bauer AJ: Molecular genetics of thyroid cancer in children and adolescents. Endocrinol Metab Clin North Am 46:389-403, 2017 28476228 10.1016/j.ecl.2017.01.014

[5] Paulson VA Rudzinski ER Hawkins DS: Thyroid cancer in the pediatric population. Genes (Basel) 10:723, 2019 31540418 10.3390/genes10090723 PMC6771006

[6] Mostoufi-Moab S Labourier E Sullivan L : Molecular testing for oncogenic gene alterations in pediatric thyroid lesions. Thyroid 28:60-67, 2018 29108474 10.1089/thy.2017.0059 PMC5770125

[7] Lloyd RV Osamura RY Klöppel G Rosai J (eds): WHO Classification of Tumours of Endocrine Organs, Volume 10 (ed 7). Lyon, France, International Agency for Research on Cancer (IARC), 2017, pp 355

[8] Edge SB Compton CC: The American Joint Committee on Cancer: The 7th edition of the AJCC cancer staging manual and the future of TNM. Ann Surg Oncol 17:1471-1474, 2010 20180029 10.1245/s10434-010-0985-4

[9] Surrey LF MacFarland SP Chang F : Clinical utility of custom-designed NGS panel testing in pediatric tumors. Genome Med 11:32, 2019 31133068 10.1186/s13073-019-0644-8 PMC6537185

[10] Chang F Lin F Cao K : Development and clinical validation of a large fusion gene panel for pediatric cancers. J Mol Diagn 21:873-883, 2019 31255796 10.1016/j.jmoldx.2019.05.006 PMC6734859

[11] Yip L Nikiforova MN Yoo JY : Tumor genotype determines phenotype and disease-related outcomes in thyroid cancer: A study of 1510 patients. Ann Surg 262:519-525, 2015. discussion 524-525 26258321 10.1097/SLA.0000000000001420 PMC5264519

[12] Bauer AJ: Pediatric thyroid cancer: Genetics, therapeutics and outcome. Endocrinol Metab Clin North Am 49:589-611, 2020 33153669 10.1016/j.ecl.2020.08.001

[13] Pekova B Sykorova V Dvorakova S : RET, NTRK, ALK, BRAF, and MET fusions in a large cohort of pediatric papillary thyroid carcinomas. Thyroid 30:1771-1780, 2020 32495721 10.1089/thy.2019.0802

[14] Potter SL Reuther J Chandramohan R : Integrated DNA and RNA sequencing reveals targetable alterations in metastatic pediatric papillary thyroid carcinoma. Pediatr Blood Cancer 68:e28741, 2021 33009870 10.1002/pbc.28741 PMC13317003

[15] The Cancer Genome Atlas Program: https://tcga-data.nci.nih.gov

[16] Zimmerman D Hay ID Gough IR : Papillary thyroid carcinoma in children and adults: Long-term follow-up of 1039 patients conservatively treated at one institution during three decades. Surgery 104:1157-1166, 1988 3194843

[17] Kakarmath S Heller HT Alexander CA : Clinical, sonographic, and pathological characteristics of RAS-positive versus BRAF-positive thyroid carcinoma. J Clin Endocrinol Metab 101:4938-4944, 2016 27689252 10.1210/jc.2016-2620 PMC5155682

[18] Pfeifer A Rusinek D Zebracka-Gala J : Novel TG-FGFR1 and TRIM33-NTRK1 transcript fusions in papillary thyroid carcinoma. Genes Chromosomes Cancer 58:558-566, 2019 30664823 10.1002/gcc.22737 PMC6594006

[19] Pozdeyev N Gay LM Sokol ES : Genetic analysis of 779 advanced differentiated and anaplastic thyroid cancers. Clin Cancer Res 24:3059-3068, 2018 29615459 10.1158/1078-0432.CCR-18-0373 PMC6030480

[20] Schulten HJ Salama S Al-Mansouri Z : BRAF mutations in thyroid tumors from an ethnically diverse group. Hered Cancer Clin Pract 10:10, 2012 22925390 10.1186/1897-4287-10-10 PMC3434056

[21] Ortiz MV Gerdemann U Raju SG : Activity of the highly specific RET inhibitor selpercatinib (LOXO-292) in pediatric patients with tumors harboring RET gene alterations. JCO Precis Oncol 4:341-347, 2020 10.1200/PO.19.00401 PMC7450975 32923911

[22] Wirth LJ Sherman E Robinson B : Efficacy of selpercatinib in RET-altered thyroid cancers. N Engl J Med 383:825-835, 2020 32846061 10.1056/NEJMoa2005651 PMC10777663

[23] Groussin L Clerc J Huillard O: Larotrectinib-enhanced radioactive iodine uptake in advanced thyroid cancer. N Engl J Med 383:1686-1687, 2020 33085869 10.1056/NEJMc2023094

